# Glioblastoma multiforme in Klippel-Trenaunay-Weber syndrome: a case report

**DOI:** 10.1186/s13256-015-0555-2

**Published:** 2015-04-17

**Authors:** Tevfik Yilmaz, Ulas Cikla, Alice Kirst, Mustafa K Baskaya

**Affiliations:** Department of Neurological Surgery, School of Medicine and Public Health, University of Wisconsin, 750 Highland Avenue, Madison, WI USA

**Keywords:** Klippel-Trenaunay-Weber syndrome, Glioblastoma multiforme, Congenital vascular anomalies, Bleeding

## Abstract

**Introduction:**

Klippel-Trenaunay-Weber syndrome (KTWS) is a rare syndrome in which patients usually present with cutaneous hemangiomas, venous varicosities, and bone and soft tissue hypertrophy of the affected limb. Intracranial lesions in patients with KTWS are extremely rare, and are generally reported as single cases in the literature. We describe a rare case, where a patient with KTWS was found with a hemorrhagic grade IV astrocytoma. Although central nervous system abnormalities such as intracranial aneurysms and cerebral and spinal cord cavernomas have been described in patients with KTWS, to the best of our knowledge, this is the first report of an association between glioblastoma multiforme (grade IV astrocytoma) and KTWS in the English-language medical literature.

**Case presentation:**

A 61-year-old white Caucasian man with a history of KTWS presented with seizures. Left upper and lower extremity hypertrophy, left foot, leg and ear gigantism and left-sided abdominal capillary hemangiomas were noted in the physical examination. Cranial computed tomography (CT) and magnetic resonance imaging (MRI) were obtained, showing a heterogeneous lesion in the cingulate gyrus, with peripheral and central areas of T1 hyperintensity and layering T2 hypointensity consistent with a hemorrhage. A right parasagittal frontal craniotomy was performed with an interhemispheric approach. We had difficulty controlling the bleeding with bipolar electrocautery during surgery and finally were able to stop the bleeding using surgicel and gelfoam. Postoperative cranial CT and MRI scans showed intraparenchymal hemorrhage centered within the medial right frontal lobe. There was no increase in hematoma size in consecutive CT scans.

**Conclusions:**

Co-occurrence of vascular abnormalities with KWTS should be taken into consideration to avoid perilous preoperative and postoperative complications.

## Introduction

Klippel-Trenaunay-Weber syndrome (KTWS) is a congenital vascular disorder characterized by cutaneous capillary hemangiomas (port-wine stains), venous varicosities, and osseous and soft tissue hypertrophy. Many theories have been developed since the discovery of KTWS; however, the etiology of the syndrome is still unknown. The general assumption outlines a polygenic etiology involving defects in angiogenesis and growth regulation of vessels and tissue [1]. Intracranial lesions in patients with KTWS are extremely rare, and are generally reported as single cases in the literature [2-6]. Astrocytoma grade IV is the most common malignant primary brain tumor in adults. Although many KTWS-associated neuropathologic comorbidities have been described in the literature, to our knowledge astrocytoma grade IV has not yet been reported as one of these comorbidities. This manuscript describes a rare case, where a patient with KTWS was found with a hemorrhagic astrocytoma grade IV. Necessary preparatory steps should be taken to avoid complications caused by vascular pathologies that may co-exist with pathologies of KTWS.

## Case presentation

**Figure 1:**
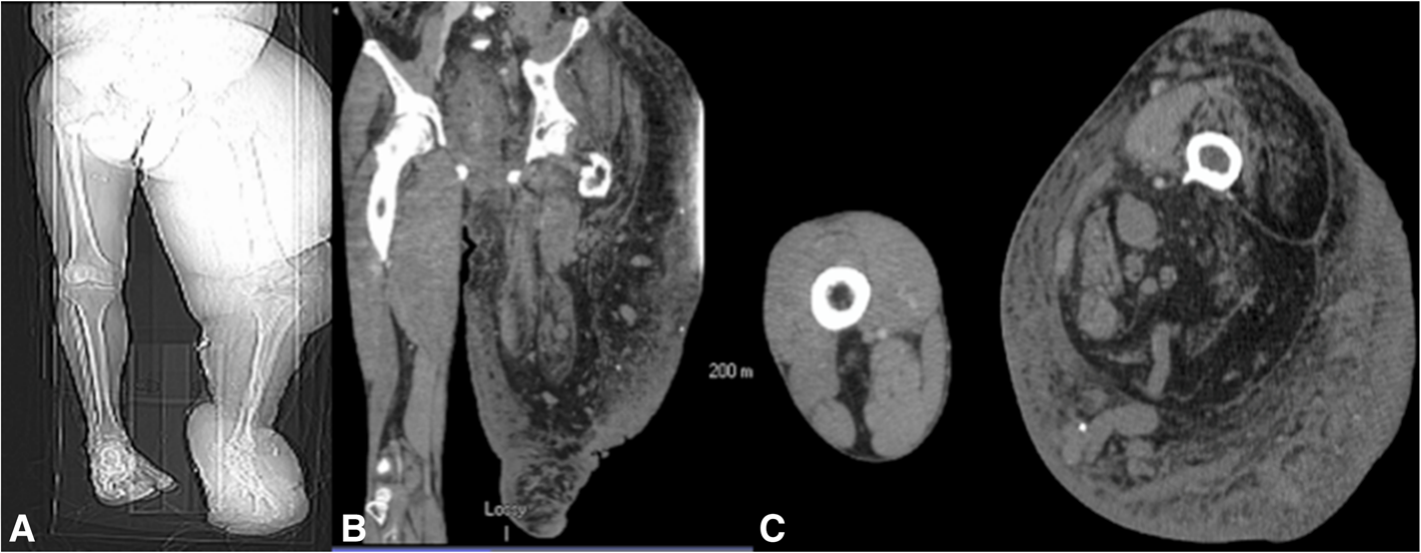
Pilot image **(A)** and the computed tomography scan with coronal **(B)** and axial **(C)** cuts of the left lower extremity showing hypertrophy of the left leg.

**Figure 2:**
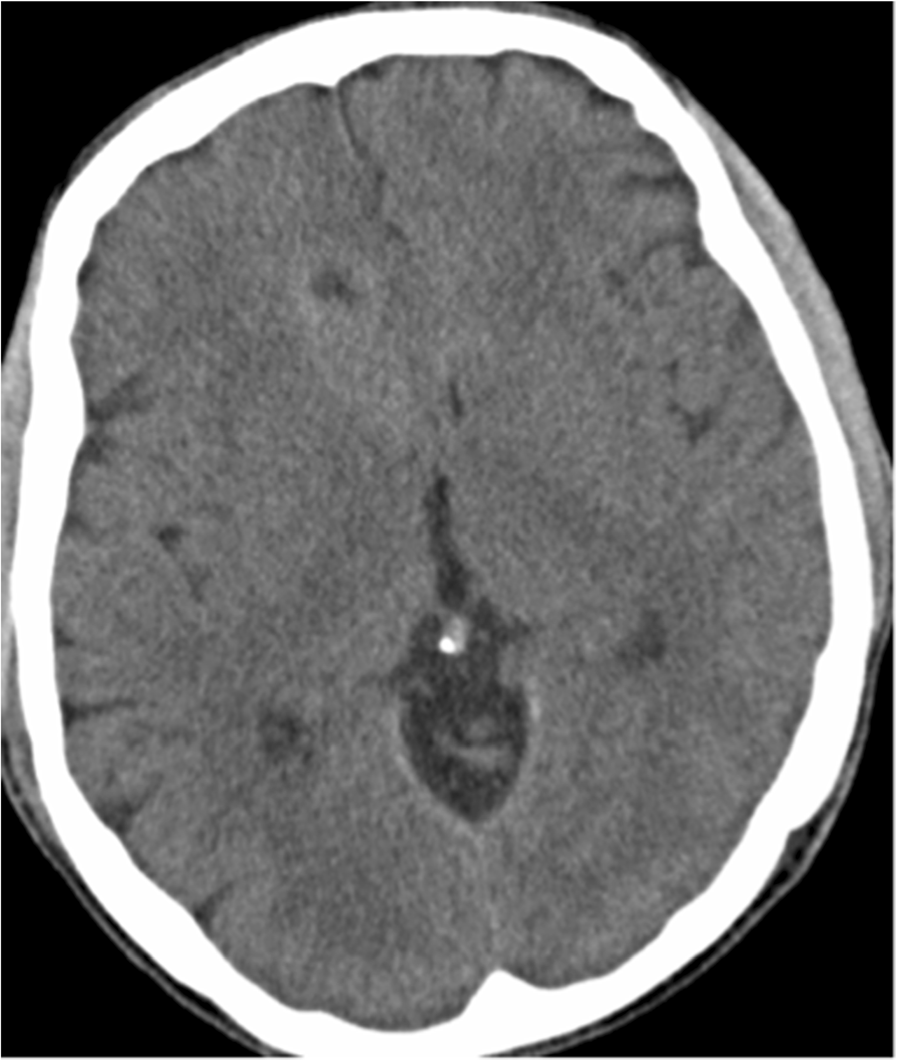
Preoperative non-contrast axial computed tomography showing a hypodense lesion with peripheral bleeding in the right anterior cingulate gyrus.

**Figure 3:**
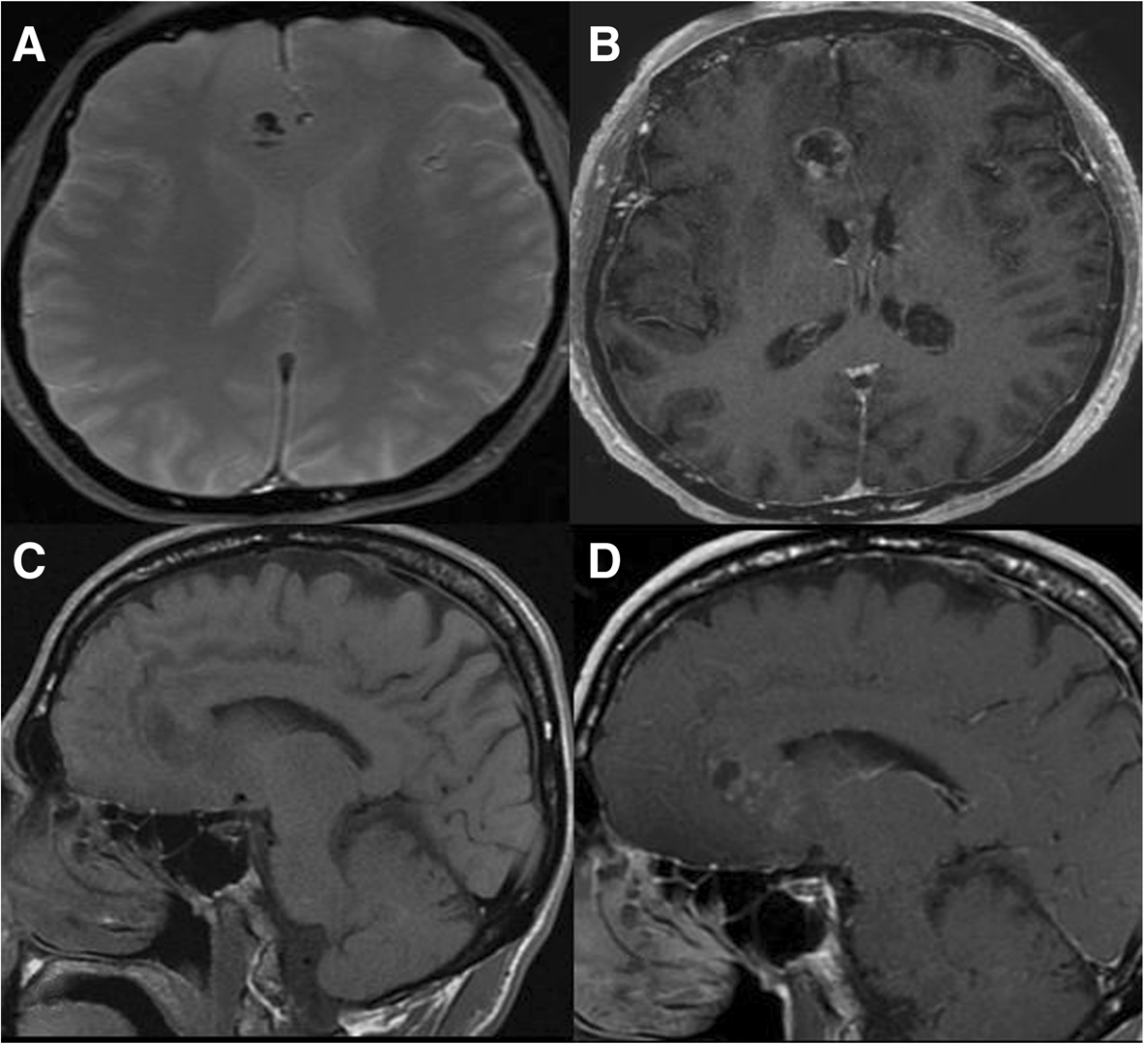
Axial echo gradient **(A)** and T1 postcontrast **(B)** and sagittal precontrast **(C)** and postcontrast **(D)** magnetic resonance imaging scans showing peripheral contrast uptake and a hemorrhagic heterogeneous lesion located in the right cingulate gyrus.

**Figure 4:**
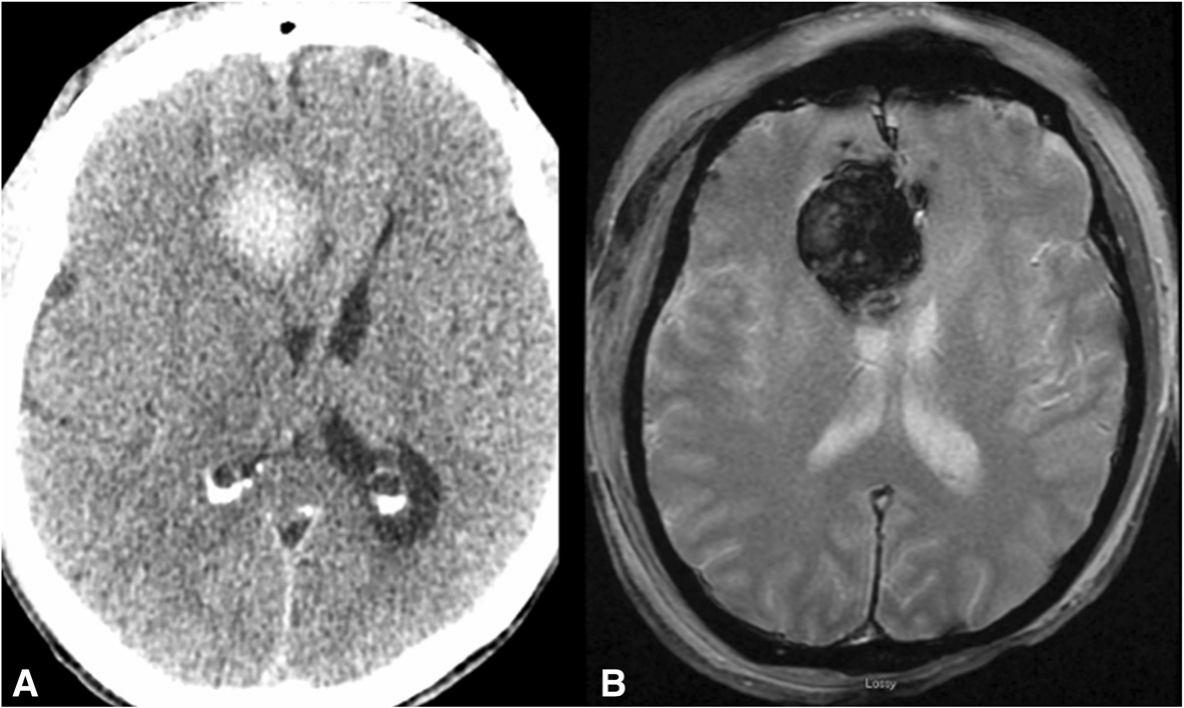
Postoperative imaging including computed tomography scan **(A)** and T2 axial **(B)** magnetic resonance imaging scan showing expansile postoperative hemorrhage.

A 61-year-old white Caucasian man with history of KTWS presented with seizures. Our patient had been diagnosed with KTWS at the age of seven after an episode of rectal bleeding. Upon neurological examination, our patient was able to follow commands and move all extremities, and exhibited mild left drift. Left upper and lower extremity hypertrophy, left foot, leg and ear gigantism and left-sided abdominal capillary hemangiomas were noted upon physical examination (Figure 1A, B, C). A head computerized tomography (CT) scan without contrast was obtained, showing a hypodense lesion with peripheral bleeding in the anteromedial right frontal lobe (Figure 2). Cranial magnetic resonance imaging (MRI) showed a heterogeneous lesion in the cingulate gyrus, with peripheral and central areas of T1 hyperintensity and layering T2 hypointensity consistent with the hemorrhage. There was minimal rim enhancement along the inferior margin of the hemorrhage (Figure 3A, B, C, D). A right parasagittal frontal craniotomy was performed with an interhemispheric approach. A hemorrhagic mass was identified and operative microscopic impression was consistent with malignant intrinsic brain tumor. Although there was no extraordinarily excessive bleeding more than usually seen in high-grade gliomas during resection of the mass, as we resected the mass and reached relatively normal-looking white matter, we encountered more bleeding from the white matter. We had a hard time controlling the bleeding with bipolar electrocautery and finally were able to stop the bleeding with surgicel and gelfoam. Platelets and plasma were transfused during surgery and during the postoperative period. Postoperative cranial CT and MRI scans showed intraparenchymal hemorrhage centered within the medial right frontal lobe (Figure 4A, B). There was no increase in hematoma size in consecutive CT scans. Our patient did not develop any additional neurologic deficit in his postoperative course. Our patient had rectal bleeding and the colonoscopic data showed bleeding in the distal colon and mid-sigmoid colon. In the surrounding mesentery were numerous nodular and tubular sections of soft tissue with density likely to represent enlarged vasculature and vascular malformations in this area. The process was described to be possibly related to the patient’s KTWS and the marked colonic wall thickening was possibly related to varices and vascular malformations, although it was nonspecific by imaging. Our patient was followed up and treated in line with the recommendations of the hematology and gastroenterology clinics, and he was eventually discharged after his general situation improved. Histopathology was reported as a grade IV astrocytoma. Our patient refused to have any adjunctive treatment and eventually died five months after the surgery due to the progression of his intracranial disease.

## Discussion

KTWS is a rare congenital phakomatosis, characterized by asymmetric limb hypertrophy, cutaneous hemangiomas and varicosities. Capillary malformations are the prevailing lesions (seen in 98% of patients), which include port-wine stains. Venous anomalies are seen in 72%, and include hypoplasia or aplasia of veins, persistence of fetal veins, varicosities, hypertrophy, tortuosity and valvular malformations. Soft tissue hypertrophy is present in 67% of cases [7]. KTWS is diagnosed when at least two of these cardinal features are met. Other signs in patients with KTWS include bleeding, thrombophlebitis, lymphatic malformation and lymphedema, hyperpigmentation, ankle ulcers and other dermal findings of varicosis [8].

Astrocytoma grade IV is the most malignant form of central nervous system tumors, and current therapies are largely ineffective at treating the cancer. Although a common brain tumor, astrocytoma grade IV in KTWS has not been previously reported.

In regard to neurovascular comorbidities, it should be noted that KTWS has been reported with accompanying central nervous system abnormalities in the literature. Aneurysmal dilatation is common, but cerebral or spinal arteriovenous malformations, persistent embryological veins, orbitofrontal varices, multiple aneurysms, meningiomatosis, epidural or vertebral hemangiomas, multiple cerebral and spinal cord cavernomas, venous malformations and duplications, hypo and aplasia have also been reported [2,5,6,9,10].

It must be noted that an abundance of these vascular pathologies leads to an increase in the surgical complication rate. It was reported preoperatively 27% of patients had a history of recurrent bleeding [7,11]. The underlying causes are venous or capillary malformations [12]. Venous malformation is the main pathology. Recurrent bleeding was very common among patients in the current review, and some patients required frequent transfusion [11]. Both sufficient intravascular access and adequate blood product reserve are of paramount importance for the management of KTWS surgery, which potentially involves massive hemorrhage. Blood loss to 18,000mL has been reported [11]. A brain hemorrhage can occur due to a capillary or venous malformation, and hypercoagulability may induce ischemic stroke [4,13]. Vascular malformations that coexist with KTWS may become a source of platelet sequestration and fibrinogen consumption, with a bleeding diathesis resulting from chronic localized intravascular consumptive coagulopathy [14]. Some authors also reported acute changes in coagulation status that develop in response to surgical procedures [15], which suggests a close postoperative monitoring of coagulation parameters in this particular patient population is necessary. A coagulation panel including partial thromboplastin time and prothrombin time is necessary to effectively monitor coagulation status in patients with KTWS both pre- and postoperatively [4].

Mason *et al*. [16] reported effective embolization in patients with noticeable coagulopathy secondary to a venous malformation following preoperative prophylaxis with platelets, cryoprecipitate or fresh-frozen plasma to facilitate thrombosis. Furthermore it has been reported that, during acute hemorrhage, tranexamic acid may stop bleeding [17]. For severe preoperative coagulopathy, it is advisable to correct coagulation parameters to reduce the risk of intra- and postoperative hemorrhage [4].

Association of KTWS with intracranial pathologies that may require surgical intervention poses a significant challenge to the managing team both preoperatively and postoperatively. In these patients, a full coagulation profile should be studied during the preoperative period. Since there is not enough experience with any specific thrombolytic agents that may stop hemorrhage, we recommended all potential agents such as platelets, cryoprecipitate, fresh-frozen plasma and tranexamic acid be readily available intraoperatively. In addition to these preoperative and perioperative precautions, postoperative care is of great importance in monitoring hemorrhagic complications.

## Conclusions

Additional vascular pathologies should be considered during the surgical planning for comorbidities accompanying KTWS, and necessary pre- and postoperative measures should be taken accordingly.

## Consent

Written informed consent was obtained from the patient for publication of this case report and accompanying images. A copy of the written consent is available for review by the Editor-in-Chief of this journal.

## Abbreviations

CT: computed tomography
KTWS: Klippel-Trenaunay-Weber syndrome
MRI: magnetic resonance imaging

## References

[1] Oduber CE, van der Horst CM, Hennekam RC. Klippel-Trenaunay syndrome: diagnostic criteria and hypothesis on etiology. Ann Plast Surg. 2008;60:217–23. 10.1097/SAP.0b013e318062abc1 18216519

[2] Boutarbouch M, Ben Salem D, Gire L, Giroud M, Bejot Y, Ricolfi F. Multiple cerebral and spinal cord cavernomas in Klippel-Trenaunay-Weber syndrome. J Clin Neurosci. 2010;17:1073–5. 10.1016/j.jocn.2009.11.013 20493710

[3] Sadiq MF, Shuaib W. Klippel-Trenaunay syndrome with intracranial arteriovenous malformation: a rare presentation. Case Rep Radiol. 2014;2014:202160. 10.1155/2014/202160 PMC3932826 24653849

[4] Star A, Fuller CE, Landas SK. Intracranial aneurysms in Klippel-Trenaunay-Weber syndromes: case report. Neurosurgery. 2010;66:1027–8. 10.1227/01.NEU.0000368392.69904.BE 20404675

[5] Djindjian M, Djindjian R, Hurth M, Rey A, Houdart R. Spinal cord arteriovenous malformations and the Klippel-Trenaunay-Weber syndrome. Surg Neurol. 1977;8:229–37. 197652

[6] Spallone A, Tcherekayev VA. Simultaneous occurrence of aneurysm and multiple meningioma in Klippel-Trenaunay patients: case report. Surg Neurol. 1996;45:241–4. 10.1016/0090-3019(95)00378-9 8638220

[7] Sreekar H, Dawre S, Petkar KS, Shetty RB, Lamba S, Naik S, et al. Diverse manifestations and management options in Klippel-Trenaunay syndrome: a single centre 10-year experience. J Plast Surg Hand Surg. 2013;47:303–7. 10.3109/2000656X.2013.766201 23710784

[8] Petzold A, Bischoff C, Conrad B. Repetitive cerebral bleeding in an adult with Klippel-Trenaunay syndrome. J Neurol. 2000;247:389–91. 10.1007/s004150050609 10896274

[9] Gourie-Devi M, Prakash B. Vertebral and epidural hemangioma with paraplegia in Klippel-Trenaunay-Weber syndrome. Case report J Neurosurg. 1978;48:814–7. 10.3171/jns.1978.48.5.0814 205634

[10] Rathbun JE, Hoyt WF, Beard C. Surgical management of orbitofrontal varix in Klippel-Trenaunay-Weber syndrome. Am J Ophthalmol. 1970;70:109–12. 10.1016/0002-9394(70)90675-6 5421636

[11] Barbara DW, Wilson JL. Anesthesia for surgery related to Klippel-Trenaunay syndrome: a review of 136 anesthetics. Anesth Analg. 2011;113:98–102. 10.1213/ANE.0b013e31821a03c2 21467557

[12] Baskerville PA, Ackroyd JS, Lea Thomas M, Browse NL. The Klippel-Trenaunay syndrome: clinical, radiological and haemodynamic features and management. Br J Surg. 1985;72:232–6. 10.1002/bjs.1800720331 2983819

[13] Brunaud V, Delerue O, Muller JP. Destee A [Klippel-Trenaunay syndrome and ischemic neurologic complications]. Rev Neurol (Paris). 1994;150:50–4. 7801041

[14] Poon MC, Kloiber R, Birdsell DC. Epsilon-aminocaproic acid in the reversal of consumptive coagulopathy with platelet sequestration in a vascular malformation of Klippel-Trenaunay syndrome. Am J Med. 1989;87:211–3. 10.1016/s0002-9343(89)80699-0 2547315

[15] Yamamoto H, Muneta T, Asahina S, Furuya K, Suzuki K. Lower leg fracture with Parkes-Weber syndrome complicated by disseminated intravascular coagulation. J Orthop Trauma. 1995;9:449–52. 10.1097/00005131-199505000-00016 8537852

[16] Mason KP, Neufeld EJ, Karian VE, Zurakowski D, Koka BV, Burrows PE. Coagulation abnormalities in pediatric and adult patients after sclerotherapy or embolization of vascular anomalies. AJR Am J Roentgenol. 2001;177:1359–63. 10.2214/ajr.177.6.1771359 11717083

[17] Katsaros D, Grundfest-Broniatowski S. Successful management of visceral Klippel-Trenaunay-Weber syndrome with the antifibrinolytic agent tranexamic acid (cyclocapron): a case report. Am Surg. 1998;64:302–4. 9544137

